# Predicting the Effect of Chemical Factors on the pH of Crystallization Trials

**DOI:** 10.1016/j.isci.2020.101219

**Published:** 2020-05-30

**Authors:** Julie Wilson, Marko Ristic, Jobie Kirkwood, David Hargreaves, Janet Newman

**Affiliations:** 1 Department of Mathematics, University of York, York, UK; 2 Collaborative Crystallisation Centre, CSIRO, Parkville, VIC, Australia; 3 Department of Chemistry, University of York, York, UK; 4 AstraZeneca, Darwin Building, Cambridge Science Park, Cambridge, UK

**Keywords:** Chemistry, Structural Biology, Crystallography

## Abstract

In macromolecular crystallization, success is often dependent on the pH of the experiment. However, little is known about the pH of reagents used, and it is generally assumed that the pH of the experiment will closely match that of any buffering chemical in the solution. We use a large dataset of experimentally measured solution pH values to show that this assumption can be very wrong and generate a model that can be used to successfully predict the overall solution pH of a crystallization experiment. Furthermore, we investigate the time dependence of the pH of some polyethylene glycol polymers widely used in protein crystallization under different storage conditions.

## Highlights

• The overall pH of crystallization solutions can be modeled
• The model was trained and tested on a set of more than 40,000 measured pH values
• A pH value can be assigned to a non-buffered crystallization cocktail
• A 12-month stability study of polyethylene glycol suggests ways to store PEGs

## Graphical Abstract

**Figure:**
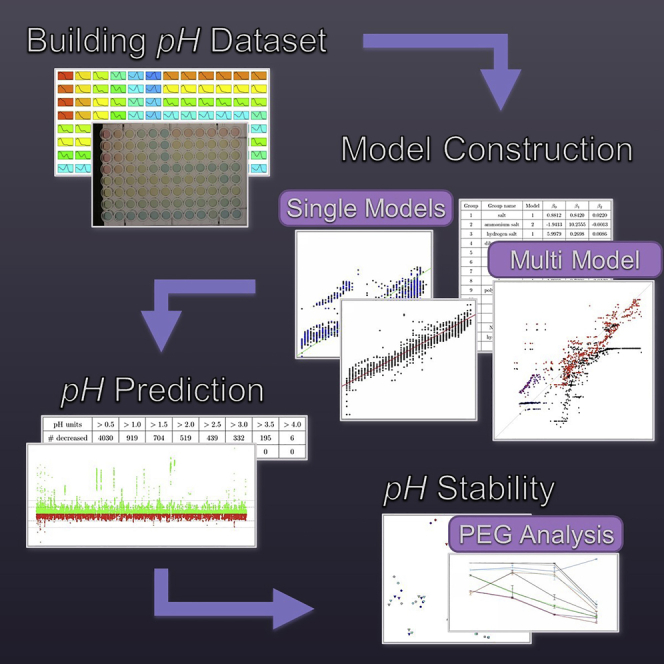

## Introduction

Macromolecular structure determination involves locating the positions, at sub-nanometre resolution, of the atoms within a protein (or other macromolecule; we will use “protein” as a general term for biological macromolecules and their complexes). Knowing the atomic structure of a protein is helpful for understanding the function of the protein, but is *essential* for structure-guided drug discovery (Thomas et al., 2017), which underpins modern development of human therapeutics. The most successful means of obtaining atomic-level structural information is through X-ray crystallography, where X-ray is diffracted through a crystalline sample of the protein of interest (McPherson and Gavira, 2013). This process requires the sample to be chemically coaxed into a sufficiently well-ordered crystalline array (a “diffraction-quality crystal”), and the production of suitable crystals is the limiting factor of this technique (Chayen, 2004). Any crystallization is a phase transition from a solution state to a solid state, and the driving force for all crystallization is supersaturation (Nývlt, 1968). In protein crystallization there is requirement not only for supersaturation but also for stabilization, as the protein's three-dimensional fold needs to be maintained during the crystallization process. However, even if the protein remains well folded it may not crystallize. Current state of the art in protein crystallization is essentially a trial-and-error search through a huge chemical landscape (Newman et al., 2007). Each point on the chemical landscape is a possible crystallization cocktail (or crystallization condition) and consists of a mixture of one or more chemicals: usually some combination of a polymer, a salt, and a buffering agent. The trial-and error-approach is used as, with rare exception (Asherie et al., 2009), there is insufficient understanding of protein crystallization to explain, let alone predict, under which conditions a protein will crystallize and why those conditions work (Cudney, 1999, Ruben, 2014).

Unlike the crystallization of small molecules, protein crystallization tends to be highly sensitive to changes in the pH of the crystallization condition (McPherson, 1995, Judge et al., 1999). The pH sensitivity of protein crystallization is due the inclusion of ionizable side chains in the protein chain. This in turn gives the protein molecule an overall charge, and the surface charge will depend on the ionization state of surface residues. The solubility of the protein will depend on the charged surface; the protein will be least soluble at a pH equal to its pI, or when the protein is overall uncharged (Tanford, 1963). Furthermore, the charged state of the surface amino acid residues of the protein influence the balance between the folded and unfolded states of the protein (Pace et al., 2000). In particular, of the 20 amino acids normally incorporated into proteins, 5—aspartic acid, glutamic acid, lysine, arginine and histidine—have side chains with pK_a_s that might be influenced by the pH of the crystallization cocktail. Furthermore, protein active sites often contain residues with ionizable side chains, so an understanding of the conditions in which a protein is crystallized can provide insight into the interpretation of details of the active site; a protein active at high pH may not reveal physiologically relevant details in the active site if crystallized at pH 4, for example.

The growth of a protein crystal from a saturated solution of the protein is a phase transition and logically must be dependent on the physical properties of the system (e.g., temperature, viscosity, dielectric, pH, etc.). What is less obvious is that the physical properties of the crystallization system are also likely to modulate the packing of the molecules in the solid (crystalline) state. There is little literature on the nature of crystal contacts and the system properties; some of the more relevant includes recent work from the area of soft matter physics, which borrows the idea of patchy particles for describing protein crystallization (Fusco and Charbonneau, 2016). However, even if there were a well-developed literature describing the relationship between solution properties and resulting crystals, there is very little known about the overall physical properties of the chemical cocktails used to prepare protein crystals. A crystallization condition is described by the concentration of its constituent chemicals rather than by its overall physical properties. For example, the pH of the condition is often assumed to be that of any buffering component of the condition—this is at best a poor approximation, as the buffering chemical is almost always found in relatively low concentration (0.01–0.1 M), and indeed, not all commercially available conditions even contain a buffering chemical.

Following (Kirkwood et al., 2015), we have developed a model for estimating the overall pH of a crystallization cocktail, using a heuristic approach that was validated by comparing the predicted final pH with an experimentally determined pH. The experimentally determined pH values were obtained from the Collaborative Crystallization Center (C3), where both initial crystallization screens and follow-on optimization screens are created in-house. Each screen is tested for quality assurance (QA) purposes with a high-throughput pH assay, where a universal dye mix is added to a small ( 10μL) aliquot of each condition in the screen (Figure S1). Through this QA assay, over 40,000 pH measurements have been made, corresponding to the same number of crystallization cocktails, and these data provide the experimental validation for the current study.

Many crystallization cocktails contain the polymer polyethylene glycol (PEG); around 70% (Abrahams and Newman, 2019) of all the conditions reported in the Protein DataBank (www.wwpdb.org; Berman et al., 2003) contain this polymer. PEG is a polymer of ethylene oxide monomers and is available in many different molecular weight ranges. PEGs are widely used in very diverse applications: for example, they are used both as a US Food and Drug Administration (FDA)-approved laxative (Fordtran and Hofmann, 2017) and as a rocket propellant (Wada et al., 2012). PEGs were introduced as protein crystallization reagents in the 1970s (McPherson, 1976) and make up a pH chemical clade. However, some PEG-containing crystallization conditions fall well outside the expected pH. PEGs are labile with light, temperature, and oxidation all thought to affect them (Cox, 1978, Glastrup, 1996, Jurnak, 1986). To determine storage conditions that would minimize the pH change seen in older PEG solutions, we investigated different temperature and lighting conditions over a 12-month period. Another possible reason for PEG-containing conditions to behave anomalously might be some intrinsic buffering behavior of the PEG itself. Although a pure PEG should have no buffering capacity, the polymerization reaction or post-processing steps might introduce some buffering capability. The inclusion of phosphate (PO_4_), a commonly used buffering ion, in PEG 3350 has been observed in C3 and by others. As PO_4_ has three pK_a_s (pK_a_1≈2.1, pK_a_2 ≈7.2, pK_a_3 ≈12.3), a PEG solution doped with PO_4_ would have some buffering around these three pH values.

**Figure 1:**
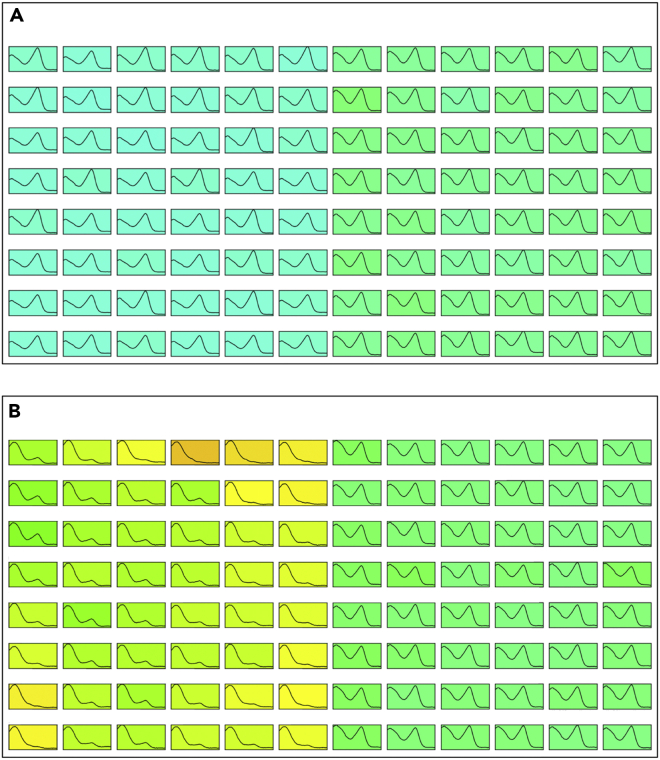
Observed Change in pH Detected using the pHUEristic Assay. pHUEristic output (see Methods) for the same screen, (A) measured when first made up and (B) measured 4 months later when the left half of the screen stopped producing crystals. For this half of the screen, containing PEG 4K, the pH had decreased by ∼2 pH units as indicated by the color change.

Chemical changes in PEGs during storage can lead to considerable changes in pH and have detrimental consequences for crystallization. An example of the effect this change in pH can have on crystallization is provided by the C3 screen shown in Figure 1. This screen contained two 48-well fine screens based around two different crystal-producing hits. Wells A1-H6 contained PEG 4K, trisand magnesium chloride-containing conditions and wells A7-H12 contained conditions consisting only of sodium formate. Initially, all conditions in the screen produced crystals. After 4 months, only the right-hand side of the screen continued to produce crystals. The pH measurements obtained for this screen after it stopped producing crystals in (the PEG-containing half of) the screen were compared with those obtained when the screen was made 4 months earlier. The half of the screen that contained PEG 4K, tris, and magnesium chloride showed a decrease in pH of around 2 pH units, whereas the other half, containing only sodium formate, showed no significant change in pH.

## Results

### Modeling pH

The dataset used to predict the pH of experiments comprised 44,639 sets of conditions from crystallization wells containing various combinations of 264 different chemicals, identified either as buffer or other (non-buffer) chemical. Some chemicals have a pH explicitly defined, but would not actually buffer a solution. To utilize the information on pH for chemicals for which the majority of occurrences were given a pH, all such chemicals were originally designated as buffers. Where modeling showed that this was not appropriate, for example, some amino acids are often given a pH value but do not behave (in the model) like buffers, chemicals were reassigned in an iterative modeling process that also determined the different chemical groups (Table 1). This process began by grouping chemicals based on assumptions about their similarity and producing regression models for each group. Subgroups with consistent but unexpected patterns were then allocated to a separate group and modeling repeated. As single chemical models were used in this stage, this was simply a case of identifying the chemicals that produced consistently large errors. In addition, different groups that produced very similar models were merged and new models built. The aim was to have as few groups as possible to avoid overfitting the data while minimizing the errors. To check for overfitting, the data in each group were divided into training and test data in the ratio 70:30 and models built using only the training data. The errors on the independent test set were then compared with those obtained from the training data. Table S2 shows the final assignment of the 264 chemicals.

**Table 1:** The Number of Times Chemicals from Each Group Are Observed

Group Number	Group Name	Number of Occurrences	Single Chemical Wells	Wells with Other Chemicals
0	Buffer	38,353	36,313^a^	1,020^b^
1	Salt	26,280	23,439	2,841
2	Ammonium salt	10,507	3,018	7,489
3	Hydrogen salt	886	117	769
4	Dihydrogen salt	508	150	358
5	PEG	30,667	4,796	25,871
6	Polyol	2,261	374	1887
7	EDTA	24	0	24
8	Polymer	37	1	36
9	Polyacrylic acid	109	0	109
10	6-Amino	59	0	59
11	Hydrochloric acid	396	12	384
12	Sodium hydroxide	503	12	491
13	Na_2_ EDTA	87	3	84
14	Hydrochloride	182	0	182
15	Arginine	27	27	0
16	No effect	3,280	482	2,798
17	Hydrogen-dihydrogen salts^c^	449	62	387

The number of wells in which a chemical from the group occurs as the only additive (non-buffer) chemical is shown as “single chemical wells.” The number of wells containing more than one additive (which may from the same group) is shown as “wells with other chemicals.” No well contained more than two different buffers.

a For buffers, the number of wells containing a single buffer is shown.

b For buffers, the number of wells containing two buffers is shown.

c Group 17 was added when it was found that the effects of hydrogen and dihydrogen salts could not be combined successfully using equation 3

A total of 367 wells were identified as containing buffer without a pH value being given, and 6,939 wells did not contain buffer. Wells containing buffer but with no buffer pH were discarded. To predict a pH for wells with no buffer we found that modeling the effect of additive chemicals on neutral pH gave good results, and therefore such wells were assigned pH 7 as the ”buffer pH.” Additive chemicals were grouped to ensure sufficient data were available for both model building and validation, and linear regression was used to relate the concentration of chemicals within a group to their effect on the buffer pH. Concentrations for all non-buffer chemicals were converted to percentage weight/volume (%w/v) to allow combinations to be considered.

### Single Chemical Models

Wells with a single non-buffer chemical were used to determine the effect of individual chemicals. Various models were investigated with the aim being to find a single best model. It was found that the effect of chemicals in most groups can be modeled to give a predicted pH value, $\hat{pH}$, as:

(Equation 1) $$\hat{pH}=\beta_{0g}+\beta_{1g}bpH+\beta_{2g}C\text{,}$$

for coefficients $\beta_{0g}$,$\beta_{1g}$, and $\beta_{2g}$determined for group *g*, where *C* is the concentration of the chemical in question and bpHis the buffer pH. However, some groups require a model of the form:

(Equation 2) $$\hat{pH}=\beta_{0g}+\beta_{1g}log_{10}\left(bpH\right)+\beta_{2g}C\text{.}$$

As Equation 2 gives significantly better results for ammonium salts and polyols, it was decided that both equations are necessary, although we have not been able to explain this chemically.

**Figure 2:**
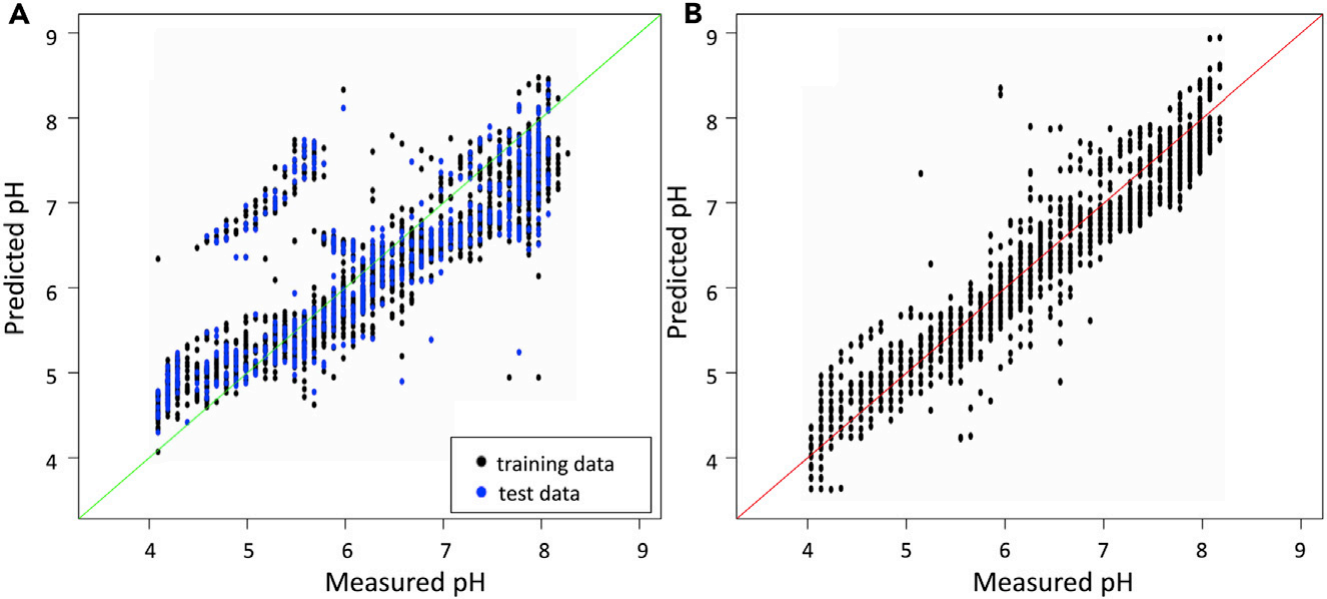
Predicted pH Values Plotted against the Measured pH for Wells Containing Ammonium Salts (A) The results from the initial ammonium salt model built including all ammonium salts, showing the separate cluster corresponding to hydrogen and dihydrogen salts. (B) The results from the model built after removing hydrogen and dihydrogen salts. Five wells with measured pH 4 and predicted pH values between pH 2 and 3.5 are not plotted.

Groups were initially formed using chemical knowledge, for example, most salts were grouped together, but as acetate salts and ammonium salts were expected to behave differently (as acetate and ammonium both affect pH, and are both somewhat volatile as ions), these were considered separately. Modeling showed that acetate salts affected the pH of an experiment in the same way as most other salts, whereas ammonium salts did behave differently. However, modeling all ammonium salts together gave poor predictions for chemicals identified as hydrogen and dihydrogen salts (Figure 2A), which were then analyzed further. In this way, some groups were combined and others were divided where common subgroups with a poor fit to the original model were identified. It can be seen that observations from the independent test dataset behave in the same way as those used for training the model. In fact, the diammonium hydrogen salts, diammonium hydrogen phosphate and diammonium hydrogen citrate, can be modeled together with other hydrogen salts, such as sodium hydrogen carbonate and dipotassium hydrogen phosphate, whereas dihydrogen salts such as ammonium dihydrogen phosphate and potassium dihydrogen phosphate require a separate dihydrogen model. Figure 2B shows the predicted pH values for ammonium salts after removal of hydrogen and dihydrogen salts from the modeling. Some very low predicted values were obtained for wells measured as pH 4 (not shown in Figure 2B), which all contain ammonium sulfate together with trisodium citrate-citric acid with a pH <3, and it could be that dye-based measurements are unreliable for such low pH values.

**Figure 3:**
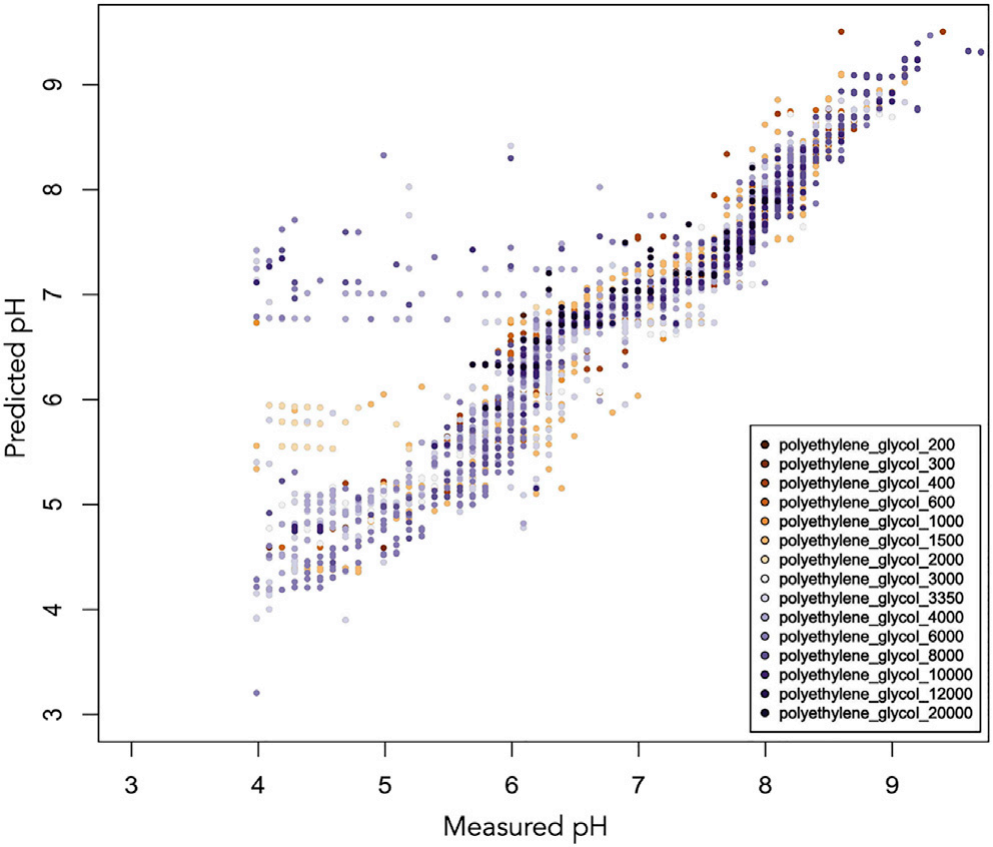
Predicted pH Values Plotted against the Measured pH for Wells Containing PEGs (with No Other Additives Except Buffer) Two subgroups with poor predictions can be seen: those with measured pH values between 4 and 5.5 and a predicted pH value between 5 and 6.5 mainly correspond to PEGs with molecular weight 1.5K or 2K, whereas those with predicted pH between 6.5 and 8.5, although the measured pH is often very acidic, correspond to PEGs with higher molecular weight (4K–8K).

**Figure 4:**
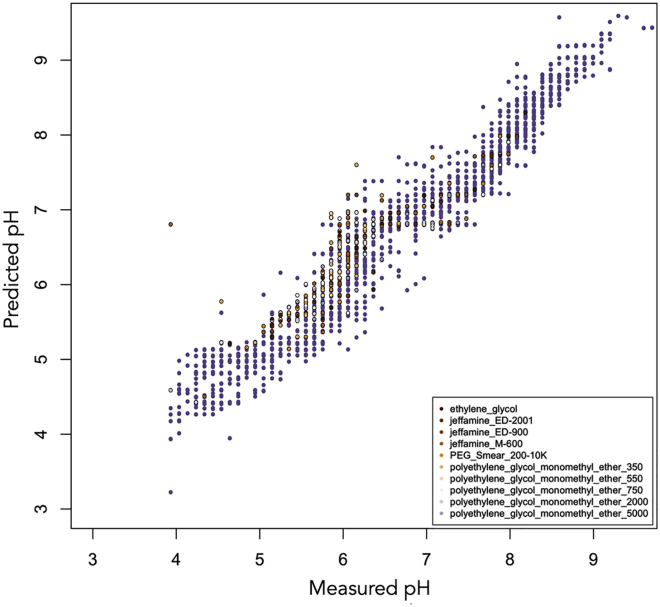
Predicted pH Values Plotted against the Measured pH for Wells Containing PEG MME, Jeffamine, or a PEG Smear The pH values were predicted using the model obtained using data from wells containing the PEGs shown in Figure 3, plotted here in lavender.

Although PEGs of different molecular weights were initially modeled separately, it was found that most behave in the same way. However, there are often cases where the predicted pH is significantly higher than the measured pH. Figure 3 shows that several wells with measured pH values between 4 and 5.5 have a predicted pH value between 5 and 6.5. Most of these wells contain PEG 2K or PEG 1500, although there are a few wells containing PEG 3350. A few wells containing PEG 3350 are among a second group of poor predictions, for which the predicted pH is between 6.5 and 8.5, although the measured pH is <7.0 and can be as low as pH 4. These wells tend to contain higher-molecular-weight PEGs, mostly PEG 4K, and also a few with molecular weights of 6K, 8K, and 10K. Although these cases could not be modeled, it was found that the PEG model obtained after ensuring none of these examples were present in the training data could be used to predict the effect of poly(ethylene) glycol monomethyl ethers (PEG MMEs) of various molecular weights as well as jeffamines, PEG smears (Chaikuad et al., 2015), and ethylene glycol (Figure 4).

Some additive chemicals, such as alcohols and organics, are rarely used as the only non-buffer chemical in a well so that insufficient data are available to produce reliable single chemical models. Furthermore, some chemicals, for example, hydrochloric acid and sodium hydroxide, used to adjust the pH, never occur as the only non-buffer chemical. However, by considering wells where they occur as the only chemical other than salt or PEG, models can be built to predict their effect on the adjusted pH obtained from the salt or PEG model as well as on the buffer pH of any wells where they do occur as the only non-buffer chemical. In this way an iterative procedure was used to provide as much data as possible for modeling different groups of chemicals.

Low-molecular-weight PEGs, including the penta-, tetra-, and triethylene glycols, which were originally considered as polyols, were found to follow the same model as much higher molecular weight PEGs. Other polyols, including sugars, required a separate mode. Dextran sulfate, together with the polyvinylpyrrolidones K15 and K25, formed another distinct cluster (the “polymer” group). Several chemicals, originally considered together as organics, were found to either have no discernible effect on the pH of a well or had very different effects from each other. In fact, 6-aminocaproic acid, polyacrylic acid, EDTA, and disodium EDTA all had to be modeled individually, and benzamidine hydrochloride, betaine hydrochloride, and guanidine hydrochloride were modeled together as hydrochlorides. It is perhaps not too surprising that these chemicals, as well as the hydrogen and dihydrogen salts, have significant and differing effects on pH. In addition to some organics, alcohols, detergents, and some amino acids were found to have negligible effect on pH, whereas other amino acids such as histidine and glycine were often assigned pH values and could therefore potentially be treated like buffers in the model. However, the pH values for these amino acids varied by more than 2 pH units and modeling was not possible where a pH was not given. Arginine, on the other hand, was found to have a large effect on pH that could be considered separately and modeled according to concentration.

All chemicals were eventually either designated as buffer or assigned to one of 16 other groups, including a group that had no effect on the buffer pH (Table 2). Table S2 shows the groups that each of the 264 different chemicals in the dataset were assigned to.

**Table 2:** Coefficients for the Linear Regression Models Obtained for Each Group

Group	Group Name	Model	$\beta_{0}$	$\beta_{1}$	$\beta_{2}$
1	Salt	1	0.8812	0.8420	0.0220
2	Ammonium salt	2	−1.9413	10.2555	−0.0013
3	Hydrogen salt	1	5.9979	0.2698	0.0086
4	Dihydrogen salt	1	1.5491	0.5185	−0.0321
5	PEG	1	1.2343	0.7989	−0.0033
6	Polyol	2	−5.0275	14.1474	−0.0061
7	EDTA	1	3.7095	0.2053	−0.0525
8	Polymer	1	1.9091	0.7398	−0.0123
9	Polyacrylic acid	1	6.7271	0.2041	−0.0102
10	6-Amino	1	3.0875	0.5195	−0.0350
11	HCl	1	−0.7778	1.2758	−1.2878
12	NaOH	1	−0.5592	1.2737	1.2428
13	Na_2_ EDTA	1	−1.3836	1.1625	0.3380
14	Hydrochloride	1	−3.7988	1.5025	−0.0312
15	Arginine	1	14.8065	−0.9341	0.0824
17	Hydrogen-dihydrogen salts^a^	1	8.4311	1.0000	−0.2641

For model 1, Equation 1 in which $\beta_{1}$is the coefficient of the buffer pH value is used; for model 2, Equation 2 in which $\beta_{1}$is the coefficient of $log_{10}$of the buffer pH value is used.

a After combining hydrogen and dihydrogen salts using Equation 6.

### Multiple Chemical Models

All supervised learning algorithms, including linear regression, can be prone to overfitting when insufficient data are available (Hawkins, 2004), and considering all possible combinations of components would result in many combinations with too few examples to be modeled accurately. We therefore chose to model the effect of multiple chemical additives by combining the pH values predicted for the individual chemicals in the cocktail. Thus the combined effects of multiple additives were determined using

(Equation 3) $$pH=-log_{10}\left(\frac{1}{M}\sum_{m=1}^{M}10^{-{\hat{pH}}_{m}}\right)\text{,}$$

where here ${\hat{pH}}_{m}$is the predicted pH of the *m*th chemical, whether or not the chemicals belong to the same group so that *M* is the total number of chemical additives. As the concentration of each chemical is taken into account when determining the individual effects on the buffer pH, via Equations 1 or 2, concentrations do not need to be considered when combining the resulting pH values, ${\hat{pH}}_{m}$, in Equation 3.

### Multiple Buffers

Initially, a combined buffer pH, denoted bpH, for wells with multiple buffers was obtained using

(Equation 4) $$bpH_{A}=-log_{10}\left(\sum_{n=1}^{N}\gamma_{n}10^{-bpH_{n}}\right)\text{.}$$

**Figure 5:**
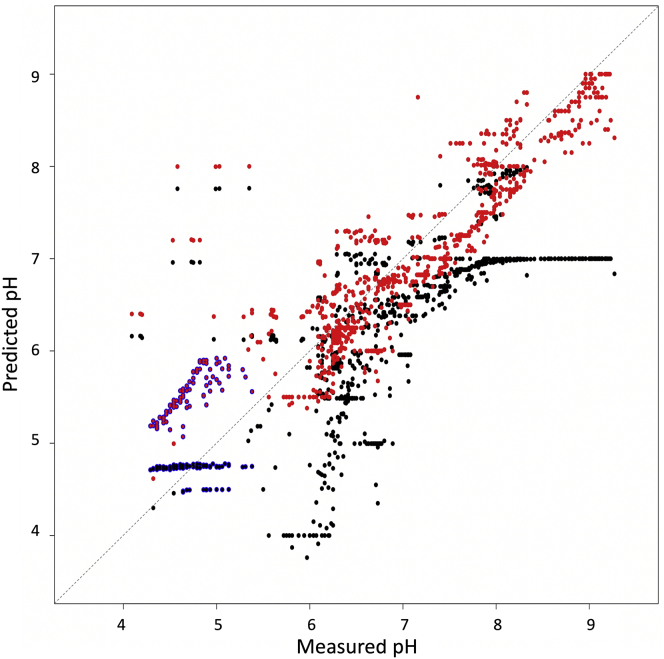
Predicted pH Values for Wells with Two Buffers. Predictions in black are obtained using Equation 4 to calculate the combined buffer $pH_{A}$_._ Predictions in red are obtained by simply averaging the two given pH values. Points outlined in blue show the predictions for a series of wells containing sodium acetate-acetic acid together with bis-tris chloride. Although the predicted pH for these wells is better when pH values are combined using Equation 4, all also contain PEG and the measured pH might have been affected by PEG degradation. The points above these with even greater prediction errors not only contain an unusual combination of many organics but also contain at least one PEG.

Here $bpH_{n}$is the pH for the *n*th buffer, *N* is the number of different buffers, and $\gamma_{n}$is the normalized concentration of the *n*th buffer. That is,

(Equation 5) $$\gamma_{n}=\frac{C_{n}}{\sum_{k=1}^{N}C_{k}}$$

where $C_{n}$is the molarity of the *n*th buffer. However, after modeling the pH as described, it was found that some of the worse errors in prediction were from wells containing two chemicals that had been assigned a pH value and were therefore both treated as buffers with a combined buffer pH calculated using Equation 4. Many wells for which the pH was predicted significantly lower than the measured pH contain sodium malonate-malonic acid, which only acts as a reasonable buffer within its buffering range. Malonate has two ionizable hydrogens and therefore two pK_a_s, one at about 2.8, and the other at 5.7. The first pK_a_ will buffer within the pH region of 1.8–3.8, and the second, between pH 4.7 and 6.7. However, outside of these pH regions, sodium malonate-malonic acid has little buffering effect at all. In particular, many conditions use sodium malonate, which has been neutralized to pH 7 and should not be considered a buffer. Figure 5 shows that considering such chemicals (those given a pH value to describe the proportions of the compound) as buffers leads to serious errors in prediction. A new model, produced to treat malonate as a chemical additive rather than a buffer after obtaining the relative proportions of sodium malonate and malonic acid using a standard curve, was unsuccessful. In fact, somewhat unsatisfactorily, the best model for combining two given pH values is to simply average the two values. Using this method for all two-buffer wells gives better predictions (after taking into account the effect of other additives) in almost all cases with the mean square error over the 1,020 wells containing two buffers reduced from 1.02 to 0.29. Figure 5 highlights a series of wells, all containing sodium acetate-acetic acid together with bis-tris chloride, for which the predictions are worse than when using Equation 4. All these wells also contain PEG (3,350, 3K, or 4K), and it could be that the measured pH has been affected by degraded PEG. Other wells with predicted pH much greater than the measured pH (with both models for combining pH values, but worse when simple averaging is used) were found to contain combinations of up to seven different organics, and it could be that the combined effects are difficult to predict, but all also contained at least one, if not two, PEGs.

### Analysis of Errors

**Figure 6:**
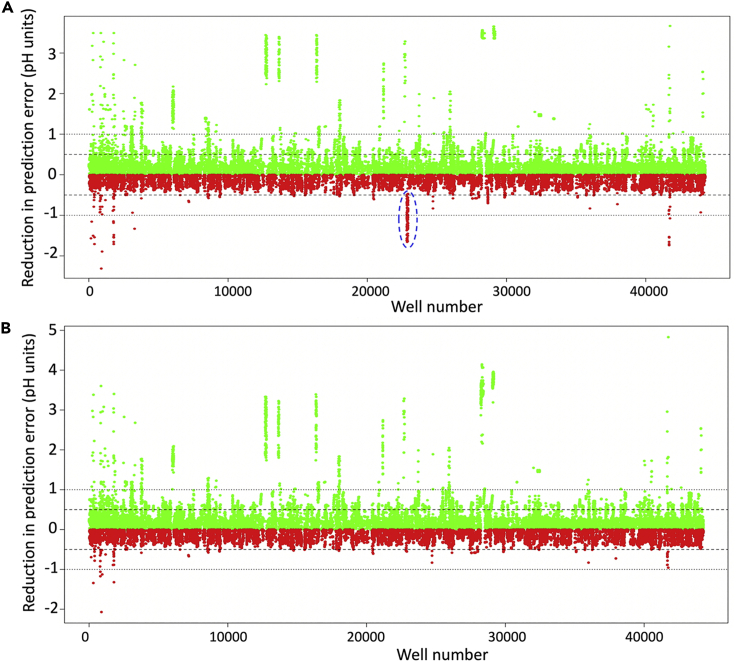
The Reduction in Error Obtained by Modeling the Effect of Additive Chemicals on the Buffer pH when Compared with Using the Buffer pH Green points indicate wells for which the predicted values have less error (from the measured pH) than the buffer pH, and red points indicate those for which the error is actually made worse. Although many errors are improved, in several cases by more than 2 pH units, most increases in error are by less than half a pH unit. Some of the worst error increases are from consecutive wells, highlighted by the blue dashed ellipse in (A). After modeling the combination of sodium dihydrogen phosphate and potassium hydrogen phosphate separately, these large errors no longer occur (B).

In some cases the pH of a well is changed very little from that of the buffer and modeling could introduce greater errors. Figure 6 shows the reduction in error obtained by modeling the effect of additive chemicals on the buffer pH compared with assuming the buffer pH as the pH of the well. Data points show the errors on the predicted values subtracted from the errors when the buffer pH is used. Thus, these values are positive when errors are reduced by modeling and negative when errors are increased. In several cases, errors are reduced by more than 2 pH units, whereas most increases in error are by less than half a pH unit. Many of the worst error increases were identified as wells containing both sodium dihydrogen phosphate and potassium hydrogen phosphate. Although good models were obtained for hydrogen salts (group 3) and dihydrogen salts (group 4) individually, these chemicals obviously interact to give a quite different effect on pH when used together. Therefore a new group was introduced to model the combined effect of these two chemicals. Modeling showed that, after combining the concentrations from the two chemicals as:

(Equation 6) hydrogen-dihydrogen=0.0353∗hydrogen-0.0509∗dihydrogen,

a regression model using the coefficients shown in Table 2 dramatically reduced the number of errors that were increased by more than 0.5 pH units. Table 3 shows the number of wells with errors decreased or increased by different amounts after modeling the effects of the 17 chemical groups when compared with using the pH of the buffer as the pH of the well. Interestingly, it was found that ammonium dihydrogen phosphate occurs in many of the wells with the most reduced errors (the high green streaks in Figure 6), suggesting that this widely used chemical has a significant effect on the buffer pH, but that this effect can be modeled.

**Table 3:** The Number of Wells with Errors that Are Increased and Decreased by Modeling when Compared with Using the Buffer pH as the pH of the Well

pH units	>0.5	>1.0	>1.5	>2.0	>2.5	>3.0	>3.5	>4.0
#Decreased	4,030	919	704	519	439	332	195	6
#Increased	154	6	1	1	0	0	0	0

**Figure 7:**
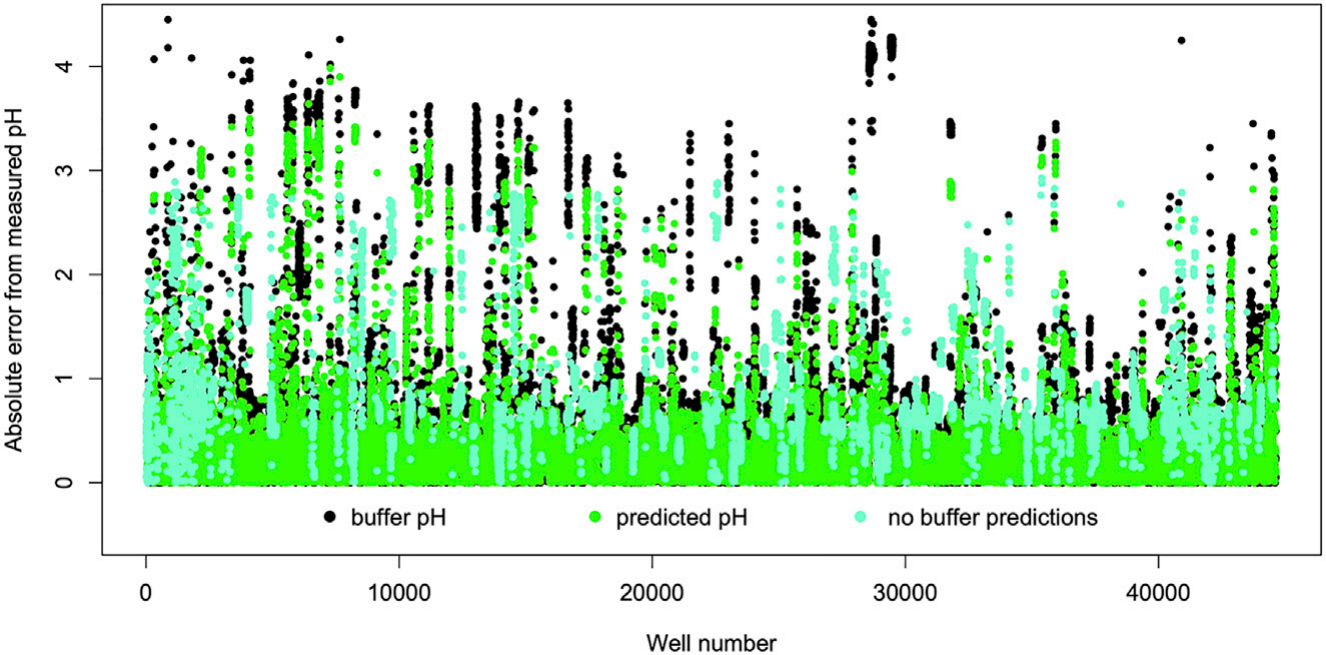
Absolute Difference Between the Measured and Predicted pH Values when Compared with the Absolute Difference Between the Measured and Buffer pH Values The pH values predicted for wells with no buffer that would otherwise have no associated pH are also shown.

The absolute differences between the measured and predicted pH values are shown in Figure 7 together with the absolute differences between the measured and buffer pH values. The errors obtained for wells with no buffer are also shown. The median absolute deviation for the wells without a buffer is 0.65 pH units, which can be compared with 0.22 pH units for wells with a buffer. However, the largest errors are of most interest and, whereas the 97th percentile of the absolute errors for the wells without a buffer at 2.65 pH units is twice that obtained for wells with a buffer (1.15 pH units), it is similar to that for the errors obtained using the buffer pH (2.22 pH units).

Some of the largest prediction errors are obtained for wells including PEG. It is interesting to note that 33 wells from the same plate containing PEG 2K with very low measured pH have large prediction errors (Figure 3). Similarly, for wells containing PEG 4K, most of the highest prediction errors were from a single plate, which could suggest that the stock solutions used had become more acidic over time. PEG is one of the most widely used reagents in crytallogenesis. Although the number of occurrences of group 5 chemicals shown in Table 1 includes jeffamines, ethylene glycol, diethylene glycol, triethylene glycol, tetraethylene glycol, and pentaethylene glycol, a staggering 30,333, or 67%, wells contain PEG (including PEG MME and PEG smear) with average molecular weights between 200 and 12K Da.

### PEG Stability over Time

**Figure 8:**
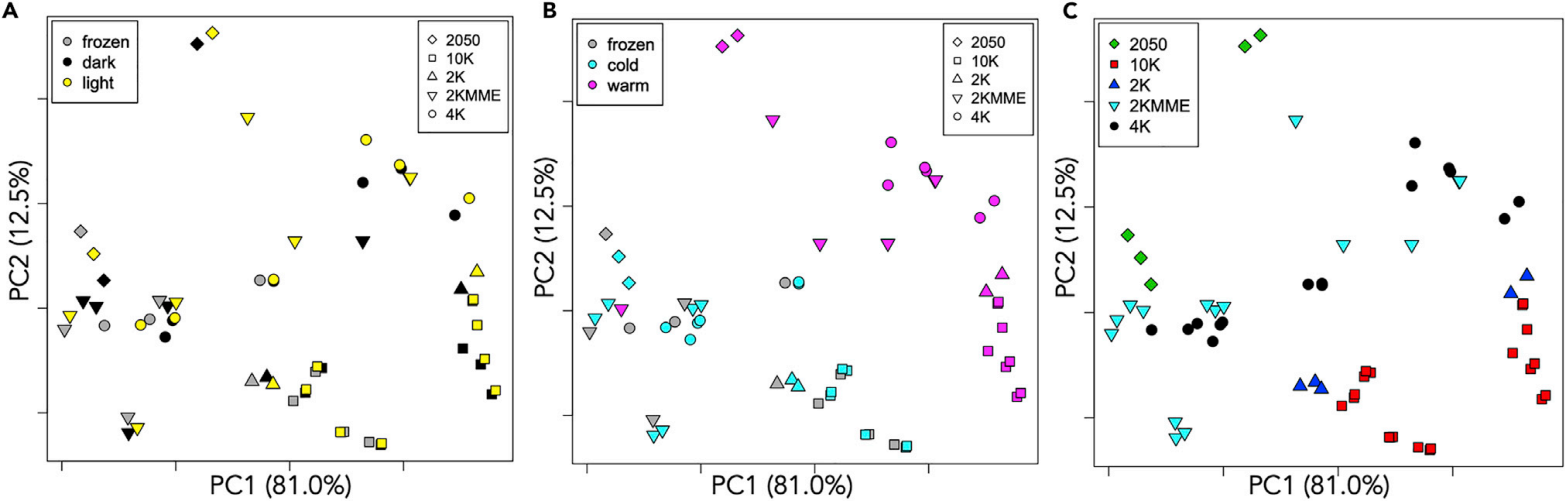
Principal-Component Analysis (PCA) Score Plots (A) There is no obvious difference between PEGs stored in the light and those stored in the dark. (B) Clear separation between PEGs stored at room temperature and those that were frozen or stored in the cold room (except for one PEG 2K MME outlier). (C) There is also some pattern with molecular weight.

Our results from PEGs stored in different conditions show that although storage temperature is important, over 12 months, there is no consistent difference between PEGs stored at 4°C and those that were frozen. Figure 8 shows a scores plot from principal-component analysis of the pH measurements obtained at 0-, 3-, 6-, and 12-month time points. Cold-stored PEGs cluster together with frozen PEGs (thawed on the day of measurement) with variance due to molecular weight, whereas PEGs stored at room temperature form a separate cluster showing the same pattern with molecular weight. One observation, a 2K PEG MME from Aldrich stored in dark, warm conditions appears to be an outlier, clustering with cold-stored PEGs. Little change in pH was observed for this PEG (obtained in solid form) until the final pair of measurements at 12 months, when a slight increase was observed, in contrast to all other PEGs stored at room temperature, which become more acidic over time. This could perhaps be due to a modulation of the aging effect due to a phosphate contamination. For PEGs stored at room temperature for 12 months, decreases of up to 2 pH units were found for 10K PEGs, up to 3 pH units for 4K PEGs, and up to 4 pH units for 2K PEGs. Line plots showing the measured pH values for PEGs from different suppliers stored at room temperature and in the cold or frozen are shown in Figures S2 and S3 respectively.

## Discussion

Crystallization screens are complex mixtures of reagents identified through trial and error. They have evolved over time with the most successful conditions perpetuating and dominating screening strategies (Jia et al., 2019). These complex mixtures are far from ideal solutions, which makes calculating their properties and behaviors impossible. However, using experimentally derived data it is possible to build a good predictive model that could be exploited to enhance our understanding of the crystallization process. Understanding how the components in crystallization mixtures behave in terms of their pH and how this varies over time allows better control over experimental conditions and improved reproducibility.

We have shown that modeling the effect of the different chemicals in the well can allow a pH value to be calculated that is closer to the measured pH than the buffer pH, sometimes by more than 3 or even 4 pH units. One condition in particular shows just how wrong the buffer pH can be as a proxy for the actual pH of the crystallization well. This condition, containing sodium dihydrogen phosphate and lithium sulfate together with CAPS (N-cyclohexyl-3-aminopropanesulfonic acid) at pH 10.5, is represented in Figure 6 as the green point in the top right-hand corner of Figure 6B) with a predicted pH of 5.67, i.e., closer to the measured pH of 5.53 by 4.83 pH units. We have tried to keep the models as simple as possible in order that new chemicals might be added easily, although obviously we cannot say how well the effect of any chemical not included in this study would be predicted.

It has been known for some time that PEGs used for protein precipitation without further purification may contain peroxides and aldehyde precursors that hydrolyze to aldehydes in aqueous solution and result in increased metal binding (Ray and Puvathingal, 1985, Jurnak, 1986). Light exposure, storage temperature, and oxygen cause changes in the chemical properties that accelerate the aging of PEGs, leading to lowered pH. The degradation of PEGs by UV light, shown by Das and Gupta (Das and Gupta, 2005), can be reduced by storing PEGs in dark-colored bottles, or covering with foil and refrigeration is preferable to storage at room temperature. Two well-known vendors of reagents for protein crystallization, Molecular Dimensions (www.moleculardimensions.com) and Hampton Research (www.hamptonresearch.com), both recommend keeping PEGs in the dark (Hampton Research Corp, 2012, Molecular Dimensions, 2019). Hampton Research also suggests that PEGs should be frozen for longer-term storage and recommends removing oxygen by flooding storage bottles with argon before freezing.

Interestingly, we did not find significant differences in pH after PEGs had been stored at 4°C for 12 months whether stored in the dark or subjected to light. However, the pH of PEGs stored at room temperature decreased by up to 4 pH units. Such effects cannot be modeled, at least not without information on age and storage conditions, but degradation can be mitigated by storing PEGs in appropriate conditions. Although our studies suggest that over the course of a year there was no difference in degradation for the PEG samples stored either cold or frozen, we cannot extrapolate these results beyond a year, and would probably suggest freezing liquid PEGs or PEG solutions for long-term storage. Similarly, although we did not find consistent differences between PEGs stored in the dark or in the light, this may not be the case for longer periods of time. However, the C3 screen shown in Figure 1 was in an opaque deep well block, heat-sealed with an aluminum seal, and therefore somewhat protected from light, but stored on a shelf by a window at around 25°C. This supports our findings that warm temperatures effect the degradation of PEGs more than light, and, as a result, all C3 blocks are now kept in the cold room for no longer than a year and PEG stocks are kept frozen.

The gradual acidification of PEG solutions observed over a year could be explained by direct oxidation of the terminal hydroxyl as described by Fishman et al. (Fishman et al., 2004) rather than through precursor contaminants (unlikely in pharmaceutical PEG 3350). Their method cites the use of chromium oxide, and the reaction is followed by ^1^H-nuclear magnetic resonance spectroscopy, matrix-assisted laser desorption/ionization-mass spectrometry, and acid-base titration. This reaction could potentially happen over a much longer timescale with atmospheric oxygen, particularly in the presence of metal ions as seen in the acidification of crystallization screens containing PEG and magnesium (e.g. Figure 1).

Different batches of PEG 3350 (and other PEGs, particularly PEG MME 2K and PEG MME 5K) used in C3 were seen to contain measurable amounts of phosphate. The same pattern of phosphate contamination was seen independently at Hampton Research (Bob Cudney, personal communication, see Table S1). Phosphate is widely used as a buffering agent in biologics, as it is non-toxic and is general regarded as safe for use in humans (https://www.fda.gov/food/generally-recognized-safe-gras/gras-substances-scogs-database). As PEG 3350 is used as an FDA-approved drug, changes in pH in the product during storage would be unacceptable. We hypothesize that the phosphate found in PEG 3350 is added during manufacturing to ensure that the pH of the product stays constant for the shelf life of the product. The inclusion of PO_4_ in some PEG formulations may have confounded this current pH analysis, but more fundamentally, can lead to confusing results in crystallization, as the combination of divalent metals in with PEG 3350 may lead to the growth of metal phosphate crystals, to the bewilderment of the crystallizer. Protein crystallization remains highly empirical with conditions often chosen based on anecdotal evidence. In the future, given enough information about the properties of crystallization solutions and their interactions with protein molecules this might change. Since the sparse matrix sampling of Jancarik and Kim (Jancarik and Kim, 1991) crystallization screens have been developed based on previously successful conditions. Such case-based reasoning takes advantage of previous experience (Jurisica and Glasgow, 2004), but introduces bias. It has been suggested that excessive consistency in the choice of chemical additives, leading to a lack of diversity in the PDB, hinders exploratory analysis (Jia et al., 2019). Even for proteins with similar properties to previously crystallized examples, deviation of the recorded pH from the true pH will affect reproducibility, especially if the protein's propensity to crystallize is over a narrow pH range. Trials will then require more than simple replication of ingredients as must have been the case for many of the >20,000 structures in the PDB solved in the past 4 years using cocktails containing PEGs. More accurate pH values that take into account the effect of the various chemicals in the crystallization cocktail could aid the reproducibility of conditions and may even allow alternative conditions with the same pH to be identified.

### Limitations of the Study

The experimental data used in this study consisted of 44,500 crystallization conditions, which contained 264 different chemicals. The commercially available screens use over 900 chemicals, demonstrating that this study does not cover all the crystallization space. As the data come from QA measurements from a working facility, rather than being created with the goal of producing a modeling training set, some of the pH groups do not have many examples, which could result in them not being well-represented in modeling. We have seen that double phosphates interact and behave differently from the way they do on their own and this could potentially be the case for some other chemicals. Furthermore, the stability studies of the PEGs followed only a subset of the PEGs used in crystallization.

### Resource Availability

#### Lead Contact

Further information and requests for resources should be directed to and will be fulfilled by the Lead Contact, Janet Newman (janet.newman@csiro.au).

#### Materials Availability

A predicted pH for a set of crystallization conditions can be obtained via the website https://phrediction.york.ac.uk.

#### Data and Code Availability

The data used in this study is available as Data S1. The source code for predicting the pH is free and available by contacting julie.wilson@york.ac.uk, and the source code for pHUEristic is free and available by contacting janet.newman@csiro.au.

## Methods

All methods can be found in the accompanying Transparent Methods supplemental file.

## Supplemental Information

Document S1. Transparent Methods, Figures S1–S3, and Tables S1 and S2

Table S3: Assignment of Individual Chemicals to Groups with Similar Effect on the pH of the Crystallization Well, Related to Table 1

Data S1. Experimental Data Used in the Study

## References

[bib1] Abrahams G., Newman J. (2019). *BLAST* ing away preconceptions in crystallization trials. Acta Crystallogr. F Struct. Biol. Commun..

[bib2] Asherie N., Jakoncic J., Ginsberg C., Greenbaum A., Stojanoff V., Hrnjez B.J., Blass S., Berger J. (2009). Tartrate chirality determines thaumatin crystal habit. Cryst. Growth Des..

[bib3] Berman H., Henrick K., Nakamura H. (2003). Announcing the worldwide protein data bank. Nat. Struct. Mol. Biol..

[bib4] Chaikuad A., Knapp S., von Delft F. (2015). Defined PEG smears as an alternative approach to enhance the search for crystallization conditions and crystal-quality improvement in reduced screens. Acta Crystallogr. D Biol. Crystallogr..

[bib5] Chayen N. (2004). Turning protein crystallisation from an art into a science. Curr. Opin. Struct. Biol..

[bib6] Cox D., Perlman D. (1978). The biodegradation of polyethylene glycols. Advances in Applied Microbiology.

[bib7] Cudney R. (1999). Protein crystallization and dumb luck. The Rigaku Journal.

[bib8] Das I., Gupta S.K. (2005). Polyethylene glycol degradation by UV irradiation. Indian J. Chem..

[bib9] Fishman A., Acton A., Lee-Ruff E. (2004). A simple preparation of PEG-carboxylates by direct oxidation. Synth. Commun..

[bib10] Fordtran J.S., Hofmann A.F. (2017). Seventy years of polyethylene glycols in gastroenterology: the journey of PEG 4000 and 3350 from nonabsorbable marker to colonoscopy preparation to osmotic laxative. Gastroenterology.

[bib11] Fusco D., Charbonneau P. (2016). Soft matter perspective on protein crystal assembly. Colloids Surf. B Biointerfaces.

[bib12] Glastrup J. (1996). Degradation of polyethylene glycol. A study of the reaction mechanism in a model molecule: tetraethylene glycol. Polym. Degrad. Stab..

[bib13] Hampton Research Corp (2012). PEG Stability: A Look at pH and Conductivity Changes over Time in Polyethylene Glycols. https://hamptonresearch.com/uploads/22.pdf.

[bib14] Hawkins D.M. (2004). The problem of overfitting. J. Chem. Inf. Comput. Sci..

[bib15] Jancarik J., Kim S.-H. (1991). Sparse matrix sampling: a screening method for crystallization of proteins. J. Appl. Crystallogr..

[bib16] Jia X., Lynch A., Huang Y., Danielson M., Lang’at I., Milder A., Ruby A., Wang H., Friedler S., Norquist A. (2019). Anthropogenic biases in chemical reaction data hinder exploratory inorganic synthesis. Nature.

[bib17] Judge R., Jacobs R., Frazier T., Snell E., Pusey M. (1999). The effect of temperature and solution pH on the nucleation of tetragonal lysozyme crystals. Biophysical J..

[bib18] Jurisica I., Glasgow J. (2004). Applications of case-based reasoning in molecular biology. Ai Mag..

[bib19] Jurnak F. (1986). Effect of chemical impurities in polyethylene glycol on macromolecular crystallization. J. Cryst. Growth.

[bib20] Kirkwood J., Hargreaves D., O’Keefe S., Wilson J. (2015). Using isoelectric point to determine the pH for initial protein crystallization trials. Bioinformatics.

[bib21] McPherson A. (1976). Crystallization of proteins from polyethylene glycol. J. Biol. Chem..

[bib22] McPherson A. (1995). Increasing the size of microcrystals by fine sampling of pH limits. J. Appl. Crystallogr..

[bib23] McPherson A., Gavira J. (2013). Introduction to protein crystallization. Acta Crystallogr. F Struct. Biol. Commun..

[bib24] Molecular Dimensions (2019). Product Description. https://www.moleculardimensions.com/products/pegs.

[bib25] Newman J., Xu J., Willis M. (2007). Initial evaluations of the reproducibility of vapor-diffusion crystallization. Acta Crystallogr. D Biol. Crystallogr..

[bib26] Nývlt J. (1968). Kinetics of nucleation in solutions. J. Cryst..

[bib27] Pace C., Alston R., Shaw K. (2000). Charge-charge interactions influence the denatured state ensemble and contribute to protein stability. Protien Sci..

[bib28] Ray W., Puvathingal J. (1985). A simple procedure for removing contaminating aldehydes and peroxides from aqueous solutions of polyethylene glycols and of nonionic detergents that are based on the polyoxyethylene linkage. Anal. Biochem..

[bib29] Ruben A. (2014). Pure, stupid luck. https://www.sciencemag.org/careers/2014/12/pure-stupid-luck.

[bib30] Tanford C. (1963). The interpretation of hydrogen ion titration curves of proteins. Adv. Protein Chem..

[bib31] Thomas S.E., Mendes V., Kim S.Y., Malhotra S., Ochoa-Montaño B., Blaszczyk M., Blundell T.L. (2017). Structural biology and the design of new therapeutics: from HIV and cancer to mycobacterial infections: a paper dedicated to John Kendrew. J. Mol. Biol..

[bib32] Wada Y., Hori K., Hasegawa K., Yagishita T., Kobayashi K., Iwasaki S., Satoh H., Nishioka M., Kimura M. (2012). Glycidyl azide polymer and polyethylene glycol as hybrid rocket fuel. Trans. Jpn. Soc. Aeronaut. Space Sci. Aerospace Technol. Jpn..

