# Supplementary material for: Predicting the Effect of Chemical Factors on the pH of Crystallization Trials
Source: iScience. 2020 May 30;23(6):101219. doi: 10.1016/j.isci.2020.101219 (PMC7298652; doi:10.1016/j.isci.2020.101219)
Supplement: Document S1. Transparent Methods, Figures S1–S3, and Tables S1 and S2 [file mmc1.pdf]

iScience, Volume *23*

Supplemental Information

Predicting the Effect of Chemical Factors on the pH of Crystallization Trials

Julie Wilson, Marko Ristic, Jobie Kirkwood, David Hargreaves, and Janet Newman

Supplemental Information

Transparent Methods

The C3 maintains a library of close to 500 stock solutions which are the basis of all of the crystallisation experiments performed in the centre. Both initial crystallisation screens and subsequent optimisation screens are manufactured "to order" using the stocks and a standard liquid handling robot(Newman, 2011). Each screen is tested for quality assurance purposes with a high-throughput pH assay, in which a dilute solution of a universal dye mix is added to a small aliquot of each condition in the screen (the assay contains 10 μL condition, 40 μL water, 50 μL 1:10 diluted dye). This is a variation of the assay described in (Newman et al., 2012; Kirkwood et al., 2015), where dye colour was determined *via* images from an RGB camera or directly from a spectrophotometer. In the work described here we used the Yamada Universal Dye mixture, which can measure pH from 4-10, and is convenient as the resultant solution colour changes approximately every pH unit(Foster and Gruntfest, 1937). The in-house analysis application 'pHUEristic' estimates the colour of the dyed solution from spectra measured from 380-780 nM in a plate reader (SPECTROstar Nano, BMGlabtech) which takes $\approx$ 1 minute. The spectra are converted to hues, which are in turn compared to the hues from spectra measured from NIST (National Institute of Health) certified pH standards. Before the resulting pH values for each crystallisation condition are inserted in the C3 database, the values are compared to both the pH of any buffer component and to the average measured pH for the condition in question, if that screen has been measured previously. A report is generated which shows the spectra, the associated hue, the buffer pH, the measured pH and the difference from the average pH value for that position in that screen, Figure S1.

Screens suspected of faults are assessed and can be re-made or discarded if necessary. The C3 database captures this information about each chemical condition in C3. The data used to model pH in this study (the chemical makeup of a crystallisation condition, and its final pH) were extracted from the C3 database. The data come from $\approx$ 450 screens measured with the assay up to mid-2018, and contain a mixture of initial screens, similar to those available commercially, and optimisation screens, where the screen consists of conditions which sample a smaller area of chemical space more densely.

For the PEG stability study, we investigated the effect of storage conditions on the pH of PEGs (and PEG monomethyl ether variants - these are labelled 'MME' in Table S1) over a period of 12 months. PEGs with average molecular weights between 400 and 10K Da were purchased from 4 different suppliers (Fluka, Aldrich, Molecular Dimensions and Hamilton Research) as shown in Table S1. Solid PEGs were made up to 25% w/v solutions and liquid PEGs diluted 50:50 with milliQ ultrapure water. A HI-2210 Bench Top pH Meter from Hannah Instruments was used to measure pH values. For each molecular weight/supplier combination, the pH of two separate aliquots was measured immediately and a further six aliquots were stored in 7 mL sterilin pots for each condition tested to allow the pH of two, previously undisturbed, pots to be measured after 3 months, 6 months and 12 months. PEGs were stored frozen ($-20°$C), in a cold room (4°) and at room temperature ($\sim 20°$, with those that were not frozen stored in both light (in a see-through plastic box) and dark (in a sealed cardboard box) conditions. See Supplementary Figures S2 and S3. We found that pH values for PEGs with average molecular weight 400 Da (from any supplier) could not be measured reliably using a pH meter.

A commercial colourometric assay (Sigma MAK307) was used to eyeball the level of phosphate in a number of the existing PEG stock solutions in the C3 laboratory. Visual inspection showed that whilst most PEG stocks showed little evidence of PO_4 contamination, the stocks of PEG 200 (made from Sigma P3015), PEG 3350 (made from Sigma P4338), PEG MME 2K (from Aldrich 202509) and PEG MME 5K (from Aldrich 81323) contained measurable levels of PO_4. Whilst the level of phosphate in the PEG 200 was low, the levels of PO_4 in the two PEG MME samples and the PEG 3350 sample were significantly greater. A more careful estimation of the phosphate concentration of the PEG 3350 was performed and gave an estimation of 1.5 mM PO_4 in C3's 50%w/v PEG 3350 stock (data not shown).

All regression modeling was carried out in the R programming environment (R Development Core Team).

Figures and Tables

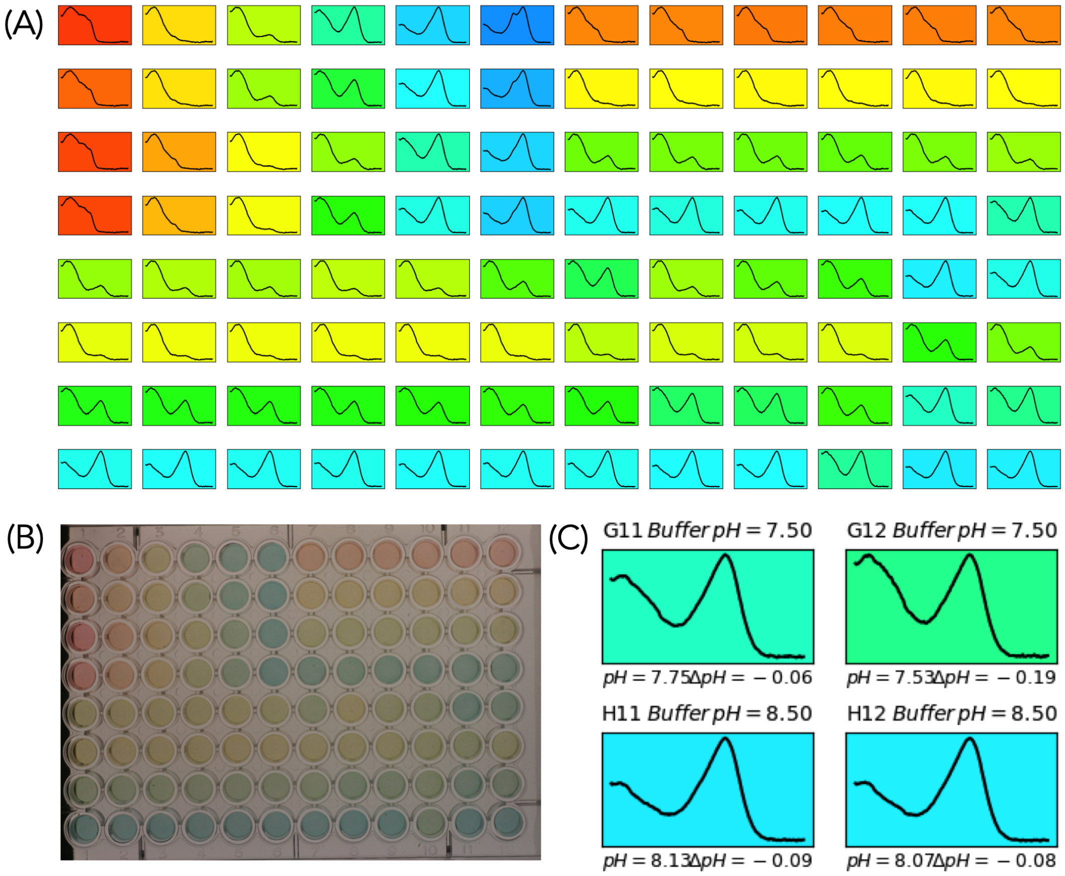

Figure S1: Measurement of pH using pHUEristic. Related to Figure 1. The pHUEristic script takes the output from a visible wavelength scan of a colour plate, and translates this into pH values. The colour plate is prepared by taking 10 μL of each condition from a 96 well screen, and adding a universal dye mixture. The script produces a report which shows the hue **(A)**reflecting the result of adding dye to the PACT screen, shown in **(B)**. Each condition in the top left hand quadrant of the PACT screen contains 25% PEG 1500, and the conditions differ by having a pH from 4 to 9, repeated with four different buffer combinations. The pH gradient is seen in the colour ramp from red (pH 4) to blue (pH 9). A zoomed view of the bottom right corner of the pH report is displayed in **(C)**, showing the measured pH (bottom left corner of each spectrum), the buffer pH (top right corner of each spectrum) and the difference between the current measured pH and the average measured pH (bottom right, this value only displays if there are other instances of the same screen that have been measured and found in the C3 database).

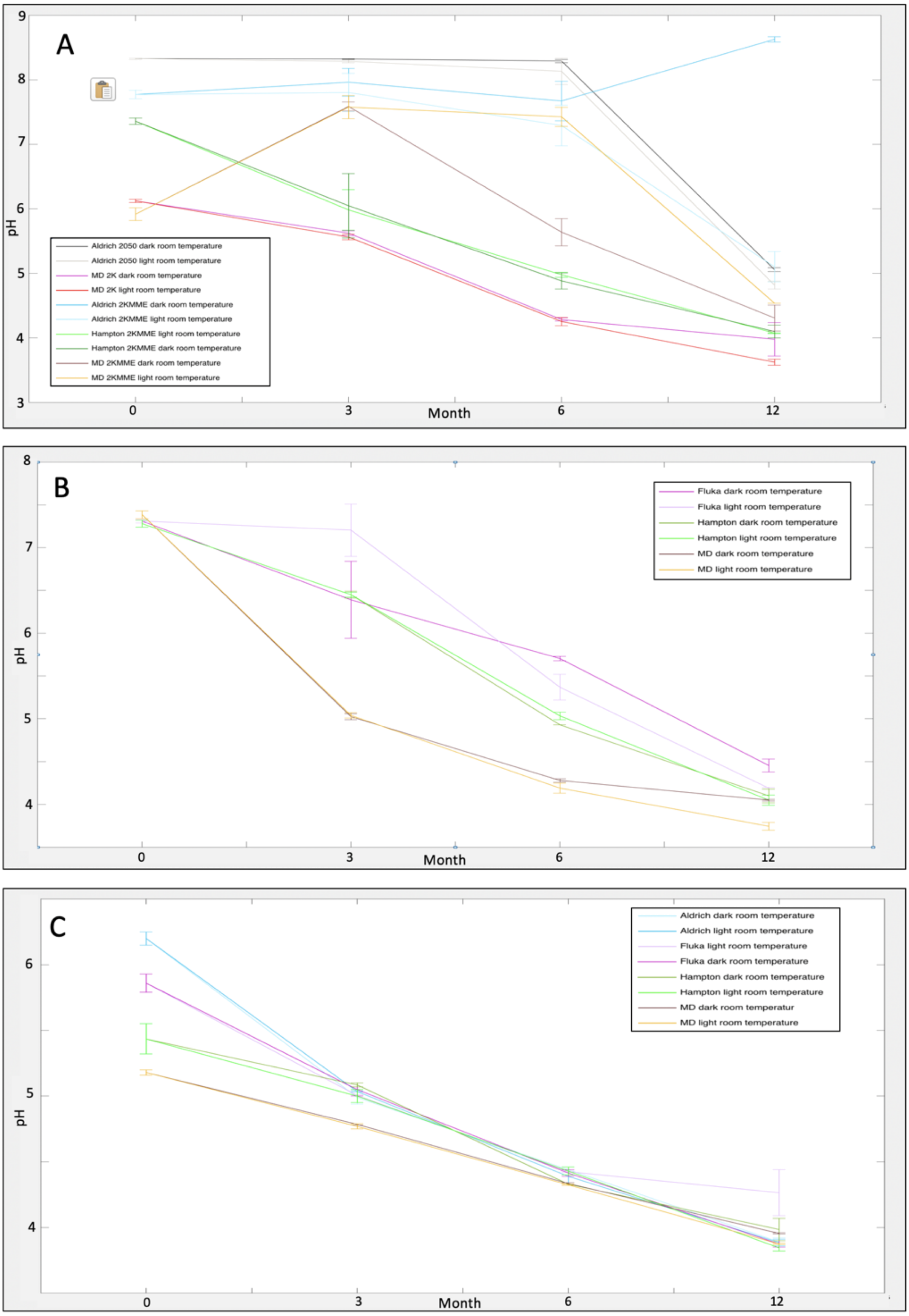

Figure S2: Measured pH values for PEGs from different suppliers stored at room temperature. Related to Figure 8. Measurements were taken at 0, 3, 6 and 12 months. The time series for PEGs of molecular weight $\sim$ 2K, 4K and 10K are shown in A, B and C respectively. Error bars show the standard deviation of the measurements from two separate aliquots.

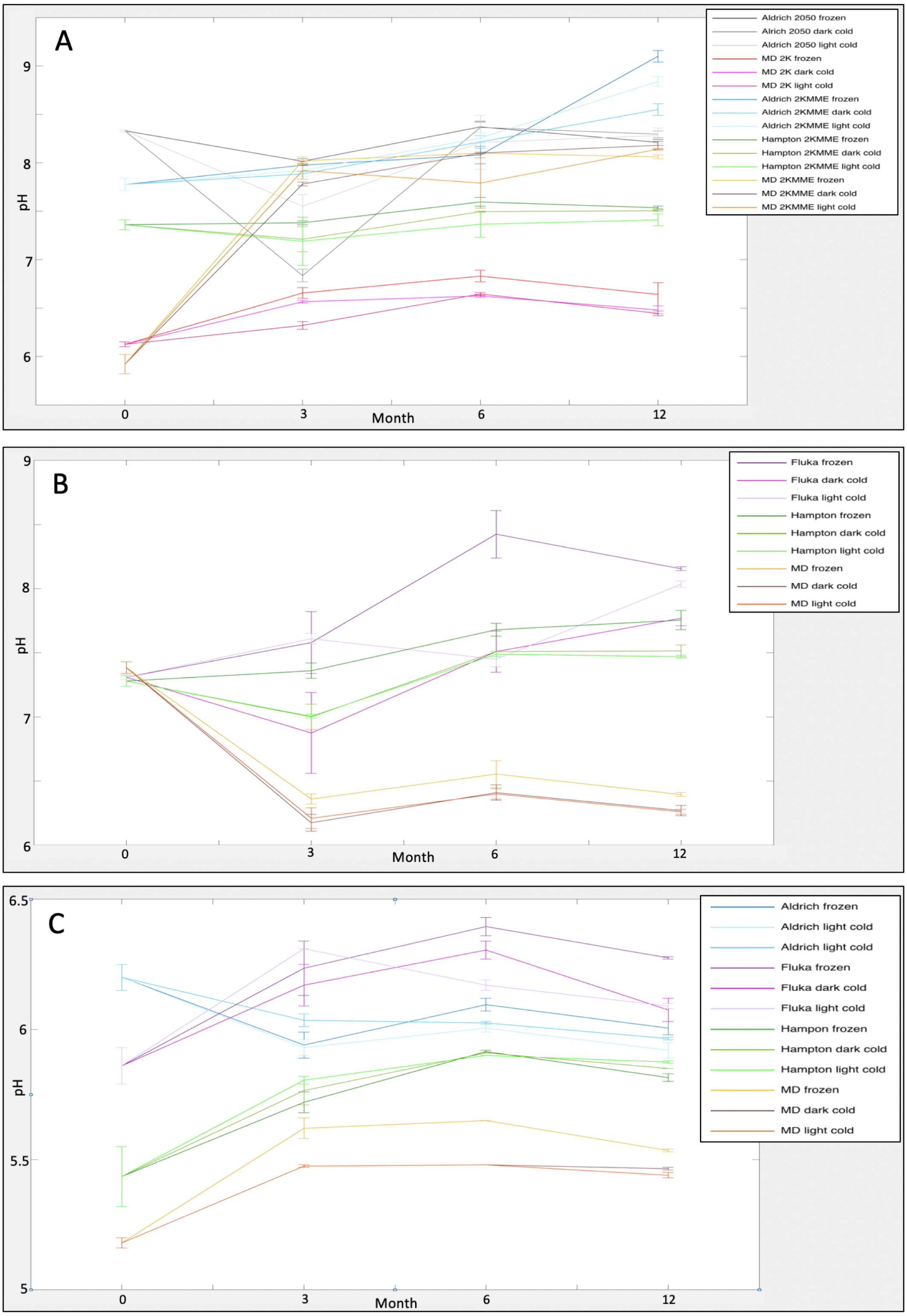

Figure S3: Measured pH values for PEGs from different suppliers stored in the cold room or frozen. Related to Figure 8. Measurements were taken at 0, 3, 6 and 12 months. The time series for PEGs of molecular weight ∼ 2K, 4K and 10K are shown in A, B and C respectively. Error bars show the standard deviation of the measurements from two separate aliquots.

Table S1: Molecular weight (M.W.), state and supplier of PEGs used to investigate storage conditions.Related to Figure 8.

M.W. (Da)	State	Supplier
400	liquid	Aldrich
2K MME	solid	Aldrich
2050	solid	Aldrich
10K	solid	Aldrich
400	liquid	Fluka
4K	solid	Fluka
10K	solid	Fluka
400	liquid	Hampton Research
2K MME	liquid	Hampton Research
4K	solid	Hampton Research
10K	liquid	Hampton Research
400	liquid	Molecular Dimensions
2K	liquid	Molecular Dimensions
2K MME	liquid	Molecular Dimensions
4K	liquid	Molecular Dimensions
10K	liquid	Molecular Dimensions

Table S2: Estimates of the PO_4 levels found in different PEGs from Hampton Research. Related to Figure 8. Phosphate levels were measured using a Biomol Green Reagent kit from Enzo Life Sciences, data kindly provided by Bob Cudney of Hampton Research.

Molecular Weight	PEG Concentration	PO_4 (mM)	PO_4 (μg/mL)
PEG 200	100% v/v	0.246	23.347
PEG 300	100% v/v	0.014	1.323
PEG 400	100% v/v	0.014	1.323
PEG 1K	50% w/v	0.011	1.033
PEG 1500	50% w/v	0.010	0.906
PEG 3350	50% w/v	0.485	46.03
PEG 4K	50% w/v	0.011	1.062
PEG 6K	50% w/v	0.012	1.175
PEG 8K	50% w/v	0.012	1.159
PEG 10K	50% w/v	0.011	1.014
PEG 20K	30% w/v	0.008	0.744
PEG MME 550	100% v/v	0.017	1.638
PEG MME 2K	50% w/v	0.011	1.049
PEG MME 5K	50% w/v	0.012	1.116

References

Foster, L. S. and Gruntfest, I. J. (1937). Demonstration experiments using universal indicators. *14*, 274.

Kirkwood, J., Hargreaves, D., O'Keefe, S. and Wilson, J. (2015). Using isoelectric point to determine the pH for initial protein crystallization trials. Bioinformatics *31*, 1444–1451.

Newman, J. (2011). One plate, two plates, a thousand plates. How crystallisation changes with large numbers of samples. Methods *55*, 73–80.

Newman, J., Sayle, R. and Fazio, V. (2012). A universal indicator dye pH assay for crystallization solutions and other high-throughput applications. Acta Crystallographica Section D Biological Crystallography *68*, 1003–1009.
